# *catena*-Poly[[[aqua­(1,10-phenanthroline-κ^2^ *N*,*N*′)zinc]-μ-acetyl­ene­dicarboxyl­ato-κ^2^ *O* ^1^:*O* ^2^] monohydrate]

**DOI:** 10.1107/S1600536812025068

**Published:** 2012-06-13

**Authors:** Mohammed Fettouhi

**Affiliations:** a Department of Chemistry, King Fahd University of Petroleum and Minerals, Dhahran, 31261, Saudi Arabia

## Abstract

In the title complex, {[Zn(C_4_O_4_)(C_12_H_8_N_2_)(H_2_O)]·H_2_O}_*n*_, the penta­coordinated Zn^II^ ion is bound to two N atoms of the 1,10-phenanthroline ligand, two O atoms from two bridging acetyl­enedicarboxyl­ate anions and a water O atom in a distorted trigonal–bipyramidal geometry. The crystal structure is characterized by polymeric zigzag chains running parallel to [2-10] and is stabilized by O—H⋯O hydrogen bonds.

## Related literature

For background to polymetallic complexes, see: Winpenny (2001 ▶); Swiegers & Malefetse (2000 ▶). For polymeric complexes based on acetyl­enedicarboxyl­ate, see: Hermann *et al.* (2011 ▶); Lin *et al.* (2011 ▶); Zheng *et al.* (2010 ▶). For a related six-coord­inate octa­hedral Zn^II^–pyridine complex with similar hydrogen-bonding inter­actions, see: Stein & Ruschewitz (2009 ▶).
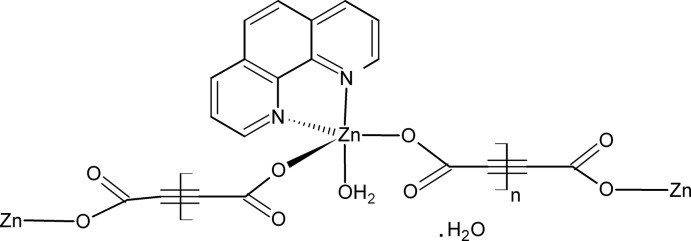


## Experimental

### 

#### Crystal data

[Zn(C_4_O_4_)(C_12_H_8_N_2_)(H_2_O)]·H_2_O
*M* *_r_* = 393.65
Triclinic, 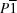

*a* = 7.9592 (8) Å
*b* = 7.9598 (8) Å
*c* = 13.3712 (13) Å
α = 105.062 (2)°
β = 101.287 (2)°
γ = 99.131 (2)°
*V* = 782.30 (13) Å^3^
*Z* = 2
Mo *K*α radiation
μ = 1.61 mm^−1^
*T* = 296 K
0.37 × 0.14 × 0.07 mm

#### Data collection

Bruker SMART APEX area-detector diffractometer
Absorption correction: multi-scan (*SADABS*; Sheldrick, 1996 ▶) *T*_min_ = 0.588, *T*_max_ = 0.896
6649 measured reflections
3398 independent reflections
3069 reflections with *I* > 2σ(*I*)
*R*_int_ = 0.018

#### Refinement

*R*[*F* ^2^ > 2σ(*F* ^2^)] = 0.041
*wR*(*F* ^2^) = 0.111
*S* = 1.14
3398 reflections
242 parameters
4 restraints
H atoms treated by a mixture of independent and constrained refinement
Δρ_max_ = 0.71 e Å^−3^
Δρ_min_ = −0.34 e Å^−3^

### 

Data collection: *SMART* (Bruker, 2007 ▶); cell refinement: *SAINT* (Bruker, 2007 ▶); data reduction: *SAINT*; program(s) used to solve structure: *SHELXS97* (Sheldrick, 2008 ▶); program(s) used to refine structure: *SHELXL97* (Sheldrick, 2008 ▶); molecular graphics: *ORTEP-3* (Farrugia, 1997 ▶); software used to prepare material for publication: *publCIF* (Westrip, 2010 ▶).

## Supplementary Material

Crystal structure: contains datablock(s) I, global. DOI: 10.1107/S1600536812025068/bt5933sup1.cif

Structure factors: contains datablock(s) I. DOI: 10.1107/S1600536812025068/bt5933Isup2.hkl

Additional supplementary materials:  crystallographic information; 3D view; checkCIF report

## Figures and Tables

**Table 1:** Hydrogen-bond geometry (Å, °)

*D*—H⋯*A*	*D*—H	H⋯*A*	*D*⋯*A*	*D*—H⋯*A*
O5—H1*W*⋯O3^i^	0.83 (2)	1.91 (2)	2.741 (4)	176 (4)
O5—H2*W*⋯O6^ii^	0.83 (2)	1.93 (2)	2.746 (4)	167 (5)
O6—H3*W*⋯O2^iii^	0.83 (2)	2.04 (3)	2.847 (5)	164 (6)
O6—H4*W*⋯O4^iv^	0.85 (2)	1.97 (2)	2.806 (4)	168 (5)

Symmetry codes: (i) 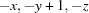; (ii) 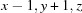; (iii) 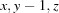; (iv) 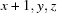.

**Fig. 1.**
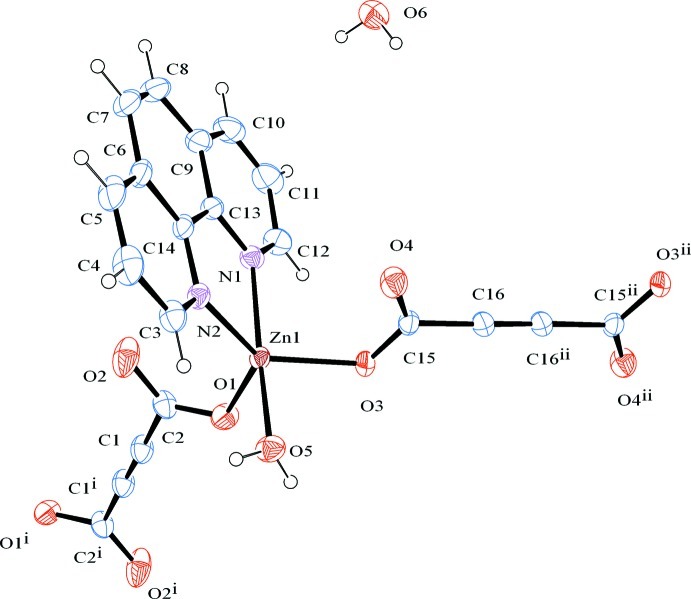
Molecular structure of a fragment of the title compound showing the atomic numbering scheme. Displacement ellipsoids are drawn at the 30% probability level (Symmetry codes: i = -x + 1,-y + 2,-z; ii = -x,-y,-z).

**Fig. 2.**
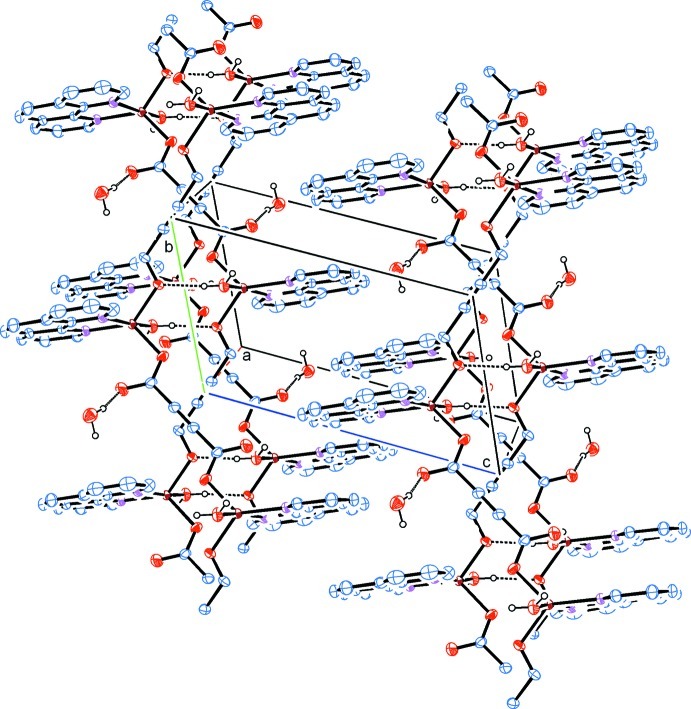
Packing diagram of the title complex showing the H-bonding interactions.

## supplementary crystallographic information

### Comment

The design and structural control of polymetallic complexes is an area of interest owing to wide potential applications (Winpenny, 2001; Swiegers *et al.*, 2000). In this context, acetylenedicarboxylate is a particularly attracting ligand as a building block of polymeric metal complexes (Hermann *et al.*, 2011; Lin *et al.*, 2011; Zheng *et al.*, 2010). I report herein on a polymeric zinc complex based on 1,10-phenanthroline and the acetylenedicarboxylate anion. The Zn(II) ion is bound to two nitrogen atoms of the 1,10-phenanthroline ligand, two oxygen atoms each belonging to an acetylenedicarboxylate anion and an oxygen atom of a water molecule (Figure 1). The geometry is distorted trigonal bipyramidal where the equatorial positions are occupied by the two carboxylate oxygen atoms and one nitrogen atom. The two acetylenedicarboxylate anions are each located on an inversion center and bridging Zn(II) ions to generate a zigzag chain structure parallel to [2 1 0] (Figure 2). The coordinated water molecule belonging to one chain interacts with a carbonyl oxygen of an adjacent chain, giving rise to eight-membered [Zn—(O···H—O)_2_—Zn] inter-chain rings with a chair configuration. One crystallization water molecule in the lattice has hydrogen bonding interactions connecting three adjacent chains by interacting with two carbonyl oxygen atoms of the first and second chain respectively in addition to a hydrogen atom of the coordinated water molecule of the third chain. A six-coordinate octahedral pyridine complex with similar hydrogen bonding interactions has been reported (Stein *et al.*, 2009).

### Experimental

Acetylenedicarboxylic acid (0.2 mmol, 0.0228 g) and 1,10-phenanthroline (0.2 mmol, 0.0397 g) were mixed in 2 ml e thanol. A clear solution was obtained upon addition of an aqueous solution (2 ml) of Zn(NO_3_)_2_.6H_2_O (0.2 mmol, 0.0595 g). The pH was then adjusted to 4.4 using NaOH (0.010 *M*) and the solution was filtered. Colorless crystals suitable for X-ray diffraction were obtained after few days by slow evaporation of the filtrate.

### Refinement

H atoms of the two water molecules were located on a difference Fourier map and refined isotropically with distance restraints of O—H = 0.84 (2) Å. All other H atoms were placed in calculated positions with a C—H distance of 0.93 Å and *U*_iso_(H) = 1.2*U*_eq_(C).

### Crystal data

**Table:**

[Zn(C_4_O_4_)(C_12_H_8_N_2_)(H_2_O)]·H_2_O	*Z* = 2
*M**_r_* = 393.65	*F*(000) = 400
Triclinic, *P*1	*D*_x_ = 1.671 Mg m^−^^3^
Hall symbol: -P 1	Mo *K*α radiation, λ = 0.71073 Å
*a* = 7.9592 (8) Å	Cell parameters from 6941 reflections
*b* = 7.9598 (8) Å	θ = 1.6–28.3°
*c* = 13.3712 (13) Å	µ = 1.61 mm^−^^1^
α = 105.062 (2)°	*T* = 296 K
β = 101.287 (2)°	Parallelepiped, yellow
γ = 99.131 (2)°	0.37 × 0.14 × 0.07 mm
*V* = 782.30 (13) Å^3^	

### Data collection

**Table:**

Bruker Smart Apex area detector diffractometer	3398 independent reflections
Radiation source: normal-focus sealed tube	3069 reflections with *I* > 2σ(*I*)
Graphite monochromator	*R*_int_ = 0.018
ω scans	θ_max_ = 27.1°, θ_min_ = 1.6°
Absorption correction: multi-scan *SADABS*; Sheldrick, 1996	*h* = −10→10
*T*_min_ = 0.588, *T*_max_ = 0.896	*k* = −10→10
6649 measured reflections	*l* = −17→17

### Refinement

**Table:**

Refinement on *F*^2^	Primary atom site location: structure-invariant direct methods
Least-squares matrix: full	Secondary atom site location: difference Fourier map
*R*[*F*^2^ > 2σ(*F*^2^)] = 0.041	Hydrogen site location: inferred from neighbouring sites
*wR*(*F*^2^) = 0.111	H atoms treated by a mixture of independent and constrained refinement
*S* = 1.14	*w* = 1/[σ^2^(*F*_o_^2^) + (0.0449*P*)^2^ + 1.0051*P*] where *P* = (*F*_o_^2^ + 2*F*_c_^2^)/3
3398 reflections	(Δ/σ)_max_ = 0.001
242 parameters	Δρ_max_ = 0.71 e Å^−^^3^
4 restraints	Δρ_min_ = −0.34 e Å^−^^3^

### Special details

**Table:**

Geometry. All e.s.d.'s (except the e.s.d. in the dihedral angle between two l.s. planes) are estimated using the full covariance matrix. The cell e.s.d.'s are taken into account individually in the estimation of e.s.d.'s in distances, angles and torsion angles; correlations between e.s.d.'s in cell parameters are only used when they are defined by crystal symmetry. An approximate (isotropic) treatment of cell e.s.d.'s is used for estimating e.s.d.'s involving l.s. planes.
Refinement. Refinement of *F*^2^ against ALL reflections. The weighted *R*-factor *wR* and goodness of fit *S* are based on *F*^2^, conventional *R*-factors *R* are based on *F*, with *F* set to zero for negative *F*^2^. The threshold expression of *F*^2^ > σ(*F*^2^) is used only for calculating *R*-factors(gt) *etc*. and is not relevant to the choice of reflections for refinement. *R*-factors based on *F*^2^ are statistically about twice as large as those based on *F*, and *R*- factors based on ALL data will be even larger.

### Fractional atomic coordinates and isotropic or equivalent isotropic displacement parameters (Å^2^)

**Table:**

	*x*	*y*	*z*	*U*_iso_*/*U*_eq_	
Zn1	0.23205 (5)	0.62227 (5)	0.17319 (3)	0.02899 (13)	
N1	0.4701 (4)	0.5448 (4)	0.2254 (2)	0.0364 (6)	
N2	0.2790 (4)	0.7239 (4)	0.3405 (2)	0.0339 (6)	
O1	0.3084 (3)	0.7402 (4)	0.0701 (2)	0.0472 (7)	
O2	0.5273 (5)	0.9248 (5)	0.1989 (2)	0.0753 (11)	
O3	0.1288 (3)	0.3764 (3)	0.06832 (19)	0.0386 (5)	
O4	0.0871 (4)	0.2719 (3)	0.2021 (2)	0.0481 (6)	
O5	−0.0117 (4)	0.6855 (4)	0.1476 (2)	0.0440 (6)	
O6	0.9009 (5)	−0.0096 (5)	0.2544 (3)	0.0570 (8)	
C1	0.4810 (5)	0.9623 (5)	0.0304 (3)	0.0432 (8)	
C2	0.4356 (5)	0.8714 (5)	0.1069 (3)	0.0417 (8)	
C3	0.1800 (6)	0.8060 (5)	0.3967 (3)	0.0439 (8)	
H3	0.0747	0.8234	0.3609	0.053*	
C4	0.2293 (7)	0.8677 (6)	0.5091 (3)	0.0588 (12)	
H4	0.1567	0.9244	0.5464	0.071*	
C5	0.3816 (7)	0.8450 (6)	0.5629 (3)	0.0622 (13)	
H5	0.4151	0.8868	0.6371	0.075*	
C6	0.4893 (6)	0.7575 (5)	0.5058 (3)	0.0488 (10)	
C7	0.6539 (7)	0.7268 (7)	0.5552 (4)	0.0673 (15)	
H7	0.6943	0.7677	0.6293	0.081*	
C8	0.7501 (6)	0.6408 (7)	0.4972 (4)	0.0674 (14)	
H8	0.8569	0.6246	0.5319	0.081*	
C9	0.6940 (5)	0.5727 (6)	0.3829 (4)	0.0519 (10)	
C10	0.7835 (6)	0.4750 (6)	0.3164 (5)	0.0639 (13)	
H10	0.8896	0.4513	0.3461	0.077*	
C11	0.7161 (6)	0.4151 (6)	0.2092 (5)	0.0620 (12)	
H11	0.7741	0.3486	0.1650	0.074*	
C12	0.5598 (5)	0.4544 (5)	0.1665 (3)	0.0469 (9)	
H12	0.5159	0.4152	0.0926	0.056*	
C13	0.5344 (4)	0.6041 (5)	0.3325 (3)	0.0366 (7)	
C14	0.4315 (5)	0.6990 (4)	0.3941 (3)	0.0366 (8)	
C15	0.0839 (4)	0.2526 (4)	0.1076 (3)	0.0341 (7)	
C16	0.0234 (5)	0.0720 (4)	0.0302 (3)	0.0356 (7)	
H1W	−0.051 (5)	0.662 (6)	0.0818 (16)	0.045 (12)*	
H2W	−0.029 (6)	0.775 (4)	0.188 (3)	0.066 (15)*	
H3W	0.795 (3)	−0.030 (8)	0.226 (4)	0.079 (19)*	
H4W	0.946 (6)	0.084 (4)	0.242 (4)	0.067 (16)*	

### Atomic displacement parameters (Å^2^)

**Table:**

	*U*^11^	*U*^22^	*U*^33^	*U*^12^	*U*^13^	*U*^23^
Zn1	0.0351 (2)	0.0266 (2)	0.02205 (19)	0.00242 (14)	0.00536 (14)	0.00573 (13)
N1	0.0358 (15)	0.0368 (15)	0.0382 (15)	0.0069 (12)	0.0095 (12)	0.0146 (12)
N2	0.0412 (15)	0.0308 (14)	0.0261 (13)	0.0017 (12)	0.0073 (11)	0.0071 (11)
O1	0.0449 (15)	0.0527 (16)	0.0448 (15)	−0.0046 (12)	0.0066 (12)	0.0288 (13)
O2	0.108 (3)	0.063 (2)	0.0341 (16)	−0.0223 (19)	0.0131 (16)	0.0070 (14)
O3	0.0461 (14)	0.0252 (11)	0.0342 (12)	−0.0001 (10)	−0.0009 (10)	0.0041 (9)
O4	0.0627 (17)	0.0410 (14)	0.0359 (14)	0.0072 (12)	0.0133 (12)	0.0057 (11)
O5	0.0486 (15)	0.0492 (16)	0.0330 (14)	0.0156 (12)	0.0073 (12)	0.0096 (12)
O6	0.068 (2)	0.057 (2)	0.0536 (18)	0.0196 (17)	0.0231 (17)	0.0201 (15)
C1	0.048 (2)	0.0378 (19)	0.040 (2)	−0.0027 (16)	0.0129 (16)	0.0109 (15)
C2	0.057 (2)	0.0356 (19)	0.0361 (19)	0.0063 (16)	0.0198 (17)	0.0130 (15)
C3	0.057 (2)	0.0379 (19)	0.0354 (19)	0.0064 (16)	0.0171 (17)	0.0071 (15)
C4	0.089 (3)	0.050 (2)	0.037 (2)	0.007 (2)	0.029 (2)	0.0050 (18)
C5	0.104 (4)	0.046 (2)	0.0228 (18)	−0.006 (2)	0.006 (2)	0.0065 (16)
C6	0.068 (3)	0.0359 (19)	0.0285 (18)	−0.0112 (18)	−0.0038 (17)	0.0108 (15)
C7	0.081 (3)	0.056 (3)	0.042 (2)	−0.015 (2)	−0.022 (2)	0.021 (2)
C8	0.053 (3)	0.066 (3)	0.068 (3)	−0.003 (2)	−0.022 (2)	0.032 (3)
C9	0.037 (2)	0.049 (2)	0.066 (3)	−0.0021 (17)	−0.0050 (18)	0.031 (2)
C10	0.037 (2)	0.061 (3)	0.100 (4)	0.015 (2)	0.006 (2)	0.040 (3)
C11	0.046 (2)	0.060 (3)	0.091 (4)	0.021 (2)	0.026 (2)	0.030 (3)
C12	0.048 (2)	0.049 (2)	0.049 (2)	0.0166 (18)	0.0177 (18)	0.0167 (18)
C13	0.0350 (17)	0.0340 (17)	0.0381 (18)	−0.0008 (14)	0.0018 (14)	0.0166 (14)
C14	0.0439 (19)	0.0299 (16)	0.0289 (16)	−0.0066 (14)	0.0013 (14)	0.0115 (13)
C15	0.0310 (16)	0.0267 (16)	0.0371 (18)	0.0055 (13)	0.0023 (13)	0.0017 (13)
C16	0.0385 (18)	0.0290 (15)	0.0378 (18)	0.0061 (13)	0.0084 (14)	0.0091 (13)

### Geometric parameters (Å, º)

**Table:**

Zn1—O1	1.992 (2)	C4—C5	1.347 (7)
Zn1—O3	2.021 (2)	C4—H4	0.9300
Zn1—O5	2.067 (3)	C5—C6	1.404 (7)
Zn1—N2	2.106 (3)	C5—H5	0.9300
Zn1—N1	2.129 (3)	C6—C14	1.400 (5)
N1—C12	1.318 (5)	C6—C7	1.435 (7)
N1—C13	1.349 (4)	C7—C8	1.335 (8)
N2—C3	1.324 (5)	C7—H7	0.9300
N2—C14	1.354 (4)	C8—C9	1.433 (7)
O1—C2	1.249 (4)	C8—H8	0.9300
O2—C2	1.227 (5)	C9—C10	1.403 (7)
O3—C15	1.267 (4)	C9—C13	1.408 (5)
O4—C15	1.227 (4)	C10—C11	1.353 (7)
O5—H1W	0.834 (19)	C10—H10	0.9300
O5—H2W	0.829 (19)	C11—C12	1.382 (6)
O6—H3W	0.826 (19)	C11—H11	0.9300
O6—H4W	0.849 (19)	C12—H12	0.9300
C1—C1^i^	1.185 (7)	C13—C14	1.435 (5)
C1—C2	1.465 (5)	C15—C16	1.477 (4)
C3—C4	1.406 (5)	C16—C16^ii^	1.171 (7)
C3—H3	0.9300		
			
O1—Zn1—O3	97.23 (11)	C4—C5—H5	120.3
O1—Zn1—O5	92.99 (11)	C6—C5—H5	120.3
O3—Zn1—O5	90.36 (11)	C14—C6—C5	117.3 (4)
O1—Zn1—N2	129.20 (11)	C14—C6—C7	118.9 (4)
O3—Zn1—N2	133.15 (10)	C5—C6—C7	123.8 (4)
O5—Zn1—N2	92.75 (11)	C8—C7—C6	121.4 (4)
O1—Zn1—N1	97.80 (11)	C8—C7—H7	119.3
O3—Zn1—N1	90.77 (11)	C6—C7—H7	119.3
O5—Zn1—N1	168.92 (11)	C7—C8—C9	122.0 (4)
N2—Zn1—N1	78.48 (11)	C7—C8—H8	119.0
C12—N1—C13	118.7 (3)	C9—C8—H8	119.0
C12—N1—Zn1	128.1 (3)	C10—C9—C13	116.8 (4)
C13—N1—Zn1	113.2 (2)	C10—C9—C8	125.6 (4)
C3—N2—C14	118.2 (3)	C13—C9—C8	117.6 (4)
C3—N2—Zn1	128.4 (3)	C11—C10—C9	120.3 (4)
C14—N2—Zn1	113.4 (2)	C11—C10—H10	119.9
C2—O1—Zn1	117.2 (2)	C9—C10—H10	119.9
C15—O3—Zn1	116.4 (2)	C10—C11—C12	119.0 (4)
Zn1—O5—H1W	108 (3)	C10—C11—H11	120.5
Zn1—O5—H2W	120 (4)	C12—C11—H11	120.5
H1W—O5—H2W	119 (5)	N1—C12—C11	123.1 (4)
H3W—O6—H4W	105 (5)	N1—C12—H12	118.4
C1^i^—C1—C2	179.1 (5)	C11—C12—H12	118.4
O2—C2—O1	125.8 (3)	N1—C13—C9	122.1 (4)
O2—C2—C1	118.4 (3)	N1—C13—C14	117.1 (3)
O1—C2—C1	115.8 (3)	C9—C13—C14	120.8 (4)
N2—C3—C4	121.9 (4)	N2—C14—C6	123.0 (4)
N2—C3—H3	119.0	N2—C14—C13	117.8 (3)
C4—C3—H3	119.0	C6—C14—C13	119.3 (3)
C5—C4—C3	120.1 (4)	O4—C15—O3	125.6 (3)
C5—C4—H4	119.9	O4—C15—C16	119.3 (3)
C3—C4—H4	119.9	O3—C15—C16	115.2 (3)
C4—C5—C6	119.4 (4)	C16^ii^—C16—C15	179.1 (5)

Symmetry codes: (i) −*x*+1, −*y*+2, −*z*; (ii) −*x*, −*y*, −*z*.

### Hydrogen-bond geometry (Å, º)

**Table:**

*D*—H···*A*	*D*—H	H···*A*	*D*···*A*	*D*—H···*A*
O5—H1*W*···O3^iii^	0.83 (2)	1.91 (2)	2.741 (4)	176 (4)
O5—H2*W*···O6^iv^	0.83 (2)	1.93 (2)	2.746 (4)	167 (5)
O6—H3*W*···O2^v^	0.83 (2)	2.04 (3)	2.847 (5)	164 (6)
O6—H4*W*···O4^vi^	0.85 (2)	1.97 (2)	2.806 (4)	168 (5)

Symmetry codes: (iii) −*x*, −*y*+1, −*z*; (iv) *x*−1, *y*+1, *z*; (v) *x*, *y*−1, *z*; (vi) *x*+1, *y*, *z*.

## References

[bb1] Bruker (2007). *SMART* and *SAINT* Bruker AXS Inc., Madison, Wisconsin, USA.

[bb2] Farrugia, L. J. (1997). *J. Appl. Cryst.* **30**, 565.

[bb3] Hermann, D., Näther, C. & Ruschewitz, U. (2011). *Solid State Sci* **13**, 1096–1101.

[bb4] Lin, J.-L., Zhu, H.-L., Zhang, J., Zhao, J.-M. & Zheng, Y.-Q. (2011). *J. Mol. Struct.* **995**, 91–96.

[bb5] Sheldrick, G. M. (1996). *SADABS* University of Göttingen, Germany.

[bb6] Sheldrick, G. M. (2008). *Acta Cryst.* A**64**, 112–122. 10.1107/S0108767307043930 18156677

[bb7] Stein, I. & Ruschewitz, U. (2009). *Z. Naturforsch. Teil B*, **64**, 1093–1097.

[bb8] Swiegers, G. F. & Malefetse, T. J. (2000). *Chem. Rev.* **100**, 3483–3537. 10.1021/cr990110s 11777430

[bb9] Westrip, S. P. (2010). *J. Appl. Cryst.* **43**, 920–925.

[bb10] Winpenny, R. E. P. (2001). *Adv. Inorg. Chem.* **52**, 1–111.

[bb11] Zheng, Y.-Q., Zhang, J. & Liu, J.-Y. (2010). *CrystEngComm*, **12**, 2740–2748.

